# Utilization Patterns of IV Iron and Erythropoiesis Stimulating Agents in Anemic Chronic Kidney Disease Patients: A Multihospital Study

**DOI:** 10.1155/2012/248430

**Published:** 2012-04-19

**Authors:** Avani D. Joshi, David A. Holdford, Donald F. Brophy, Spencer E. Harpe, Darcy Mays, Todd W. B. Gehr

**Affiliations:** ^1^Global Heath Economics and Outcomes Research, Abbott Laboratories, North Chicago, IL 60064, USA; ^2^Department of Pharmacotherapy and Outcomes Science, Virginia Commonwealth University School of Pharmacy, P.O. Box 980533, 1112 East Clay Street, Richmond, VA 23298, USA; ^3^Department Epidemiology and Community Health, Virginia Commonwealth University School of Medicine, P.O. Box 980533, Richmond, VA 23298, USA; ^4^Statistical Sciences & Operations Research, Virginia Commonwealth University, P.O. Box 843083, Richmond, VA 23284, USA; ^5^VCU Internal Medicine, Division of Nephrology, Medical College of Virginia Campus, Virginia Commonwealth University, Richmond, VA 23298, USA

## Abstract

Intravenous (IV) iron and Erythropoiesis Stimulating Agents (ESAs) are recommended for anemia management in chronic kidney disease (CKD). This retrospective cohort study analyzed utilization patterns of IV iron and ESA in patients over 18 years of age admitted to University Health System Hospitals with a primary or secondary diagnosis of CKD between January 1, 2006 to December 31, 2008. A clustered binomial logistic regression using the GEE methodology was used to identify predictors of IV iron utilization. Only 8% (*n* = 6678) of CKD patients on ESA therapy received IV iron supplementation in university hospitals. Those receiving iron used significantly less amounts of ESAs. Patient demographics (age, race, primary payer), patient clinical conditions (admission status, severity of illness, dialysis status), and physician specialty were identified as predictors of IV iron use in CKD patients. Use of IV iron with ESAs was low despite recommendations from consensus guidelines. The low treatment rate of IV iron represents a gap in treatment practices and signals an opportunity for healthcare improvement in CKD anemic patients.

## 1. Introduction

 In the United States, Chronic kidney disease (CKD) affects approximately 26 million Americans and is the cause of significant morbidity and mortality in 1 of 9 adults [1]. Anemia is a common comorbidity of CKD–prevalent in 47% of patients who are not on dialysis [2]. Anemia of CKD results from underproduction of endogenous erythropoietin by the kidneys [3]. In patients with CKD not requiring dialysis, untreated anemia increases cardiovascular risk, hospitalization [4], all-cause mortality [5], and impaired cognitive function [1], and diminishes health-related quality of life [6] and exercise capacity [7, 8]. Heightened risk for progression of kidney failure has also been linked to untreated anemia of CKD. Thus, management of anemia in CKD patients is essential [9–11].

Erythropoiesis-stimulating agents (ESAs), such as epoetin alfa (EPO) and darbepoetin, are used to treat anemia. Use of ESAs substantially reduces the need for transfusions and therefore are a first line of therapy for anemia of CKD [12].

Despite their benefits, use of ESAs has inherent risks. ESAs have been associated with increased risk of adverse events, such as cardiovascular complications [13–16], hypertension [17], and pure red cell aplasia [18]. The US Food and Drug Administration (FDA) added a black box warning to the labeling of all epoetin and darbepoetin products advising prescribers to adjust ESA dosing to maintain the lowest hemoglobin level needed to avoid the need for red blood cell transfusion [19]. In addition, the FDA recently required all ESAs be a part of the Risk Evaluation and Mitigation Strategies (REMS) program to ensure the safe use of these drugs [20].

Adding iron to ESA regimens may reduce the dose of ESAs required to target Hb levels and therefore reduce the risk of adverse events. Iron supplementation replaces iron lost through the process of erythropoiesis stimulated by ESAs. Indeed, the Dialysis Patients Response to IV Iron with Elevated Ferritin (DRIVE I and II) studies showed iron supplementation with ESAs to be associated with higher Hb levels and fewer serious adverse events [21, 22]. Consequently, the National Kidney Foundation-Kidney Disease Outcomes Quality Initiative (NKF-KDOQI) guidelines recommend that supplemental iron be administered intravenously in hemodialysis dependent CKD patients [23]. Guidelines also recommend that nondialysis-dependent CKD patients and peritoneal dialysis dependent CKD patients receive iron orally or intravenously [23]. Ongoing administration of parenteral iron preserves levels of hemoglobin and reduces the requirement for administration of erythropoietin [15, 16, 24].

There have been no studies examining recent medication utilization patterns of IV iron in CKD patients. A single previous investigation reported trends in IV iron use among US Medicare dialysis patients [25]. The study reported an increase in the use of IV iron in ESRD patients from 1997 to 2002 with ferric gluconate and iron sucrose being the predominant form of therapy [25]. This investigation uses more recent data to quantify the rate and extent of IV iron and ESA utilization in anemic CKD patients resulting from the utilization of ESA with IV iron and without IV iron.

The primary objectives of this investigation are as follows:

1. to quantify the rate and extent of utilization of IV iron and ESAs in anemic CKD patients across teaching hospitals in the US,
2. to identify predictors of IV iron and ESA use among the domains of patient characteristics, clinical conditions, physician characteristics, hospital characteristics and treatment characteristics.

The authors hypothesize that IV iron supplementation will lead to reduction in ESA use in anemic CKD patients and that IV iron use is associated with patient characteristics, clinical conditions, physician characteristics, hospital characteristics, and treatment characteristics of anemic CKD patients.

## 2. Methods

### 2.1. Data

Data for this research came from the University HealthSystem Consortium (UHC) hospital database, a member-driven alliance of approximately 90% of the nonprofit academic medical centers in the United States. For this study, UHC's Clinical Resource Manager (CRM) database was used to gather data from hospital discharge summaries and Uniform Billing-92 data. Inpatient records from the database provided primary and secondary diagnoses (in *International Classification of Diseases, 9th Revision, Clinical Modification *[ICD-9-CM] format), inpatient procedure codes (in ICD-9-CM format), patient demographic information (age, race, gender, primary and secondary insurer), and hospital demographic information (bed size and geographical location). The database also provided admission and discharge dates as well as information on comorbidities, severity of illness, and physician specialty.

### 2.2. Study Population

The data warehouse was electronically queried for patients with *Chronic Kidney Disease *using ICD-9-CM codes. Eligible patients were those who were admitted to a UHC hospital with primary or secondary diagnoses of CKD who received either IV iron or ESA or both at least once during the period of January 1, 2006, and December 31, 2008.

### 2.3. Inclusion and Exclusion Criteria

Patients eligible for inclusion had to be at least 18 years of age with primary or secondary diagnosis of CKD. The patients in the treatment cohort were required to have received ESA alone or ESA + IV iron treatment during the study's time period. Patients with evidence of cancer diagnosis, chemotherapy or radiotherapy, blood transfusion, severe gastrointestinal bleeding, HIV/AIDS during the observation period were excluded to avoid including patients receiving ESA or IV iron for reasons other than anemia of CKD. Diagnoses and procedure codes used for inclusion and exclusion are described in Tables 1 and 2. This study was approved by the Virginia Commonwealth University institutional review board.

### 2.4. Statistical Analysis

Statistical analyses were performed using the Statistical Analysis Software (SAS v. 9.2) and the Predictive Analytical Software (PASW v. 17.0, previously SPSS) statistical software. A two-sided alpha of 0.05 was considered significant.

For description of IV iron and ESA use, percentages and chi-square values were calculated for the study sample and subcategories. To assess the prevalence rate of usage of ESA and IV iron from 2006 to 2008, a trend evaluation was performed for both the drugs. Differences between the study groups were calculated using *t*-tests, statistical significance set at *P* < 0.05. The days of therapy (DOTs) for ESA and IV iron therapy administered to individual patients were determined. The mean duration of therapy for each drug was calculated. The aggregate of drug use in each hospital for each year was expressed as DOTs per 100 patient-days (PDs). For example, if a patient received a single dose of a drug (ESA or IV iron) on a given day, whether or not multiple doses are usually administered, it was registered as 1 DOT. If a patient received more than 1 ESA drug (epoetin or darbepoetin) on the same day, it was counted as 1 DOT for ESA therapy. Days of hospitalization for each patient at each hospital were summed to provide total patient-days (PDs).

A clustered binomial logistic regression model using the GEE methodology was used to identify the predictors of IV iron use. The dependent variable was drug use, and the independent variables to be included in the model were (1) patient characteristics (age, race, gender, length of stay, primary payer), (2) patient clinical conditions (admission status, severity of illness, discharge status, dialysis status), (3) physician characteristics (physician specialty), (4) hospital characteristics (total hospital costs). Comparisons were considered statistically significant at *P* < 0.05. A goodness of fit test (QIC and QICC) was performed to evaluate how well the model fits the observations [26]. The goodness of fit test helped determine which of the correlation structures was more appropriate and the best subset of predictors. A Huber-White sandwich estimator (robust estimator) was used to ensure that the variances were robust [27]. Specifically, robust variances are important as they provide accurate assessments of the sample-to-sample variability of the parameter estimates even if the model is misspecified.

## 3. Results

### 3.1. Study Population

Demographic, clinical, and treatment characteristics for the study sample for the categorical variables are described in Tables 3, 4, and 5. The typical patient in the total sample was a black male over 50 years of age with an emergency admission paid for by Medicare. The average patient was not on dialysis, had an illness status rated as “major,” and was discharged or transferred alive. The typical patient was treated by an internal medicine physician, in a hospital of more than 600 beds, and more likely to receive epoetin as an ESA.

Of the 82,947 patients receiving ESAs, only 6,678 (8%) received IV iron. Univariate chi-square tests indicated significant differences for all categories of demographic, clinical, and treatment characteristics although some variables had greater practical differences than others. One difference was the age related-impact of IV iron use which increased with age. Another difference was race where whites were more likely than other racial categories to receive iron with ESAs. Iron supplementation was more likely with epoetin than darbepoetin, and iron sucrose was used much more often with ESAs than iron dextran. Univariate statistical differences in gender, primary payer, admission status, severity of illness, discharge status, dialysis status, physician specialty, bed size, and geographical region were present although they varied by a few percentage points.

### 3.2. Trends for IV Iron and ESA Use and Days of Therapy

**Figure 1:**
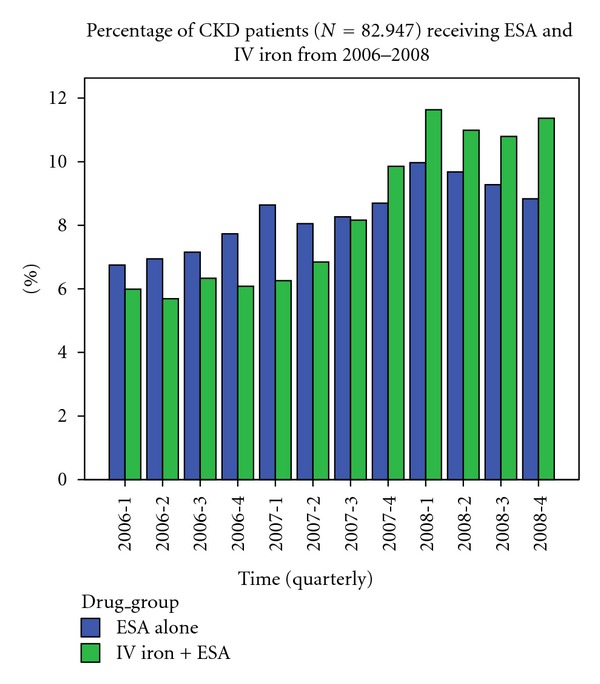
Quarterly percentages of the study population receiving ESA alone and ESA + IV iron from 2006–2008.

Figure 1 displays the usage trends for IV iron and ESA therapy in CKD patients over the study period. ESA use increased from 2006 to 2007 and then decreased after the last quarter of 2007 to 2008. In contrast, the use of IV iron therapy in CKD patients on ESA showed a small but steady increase over the two-year period. Comparing the two therapeutic groups (ESA + IV iron and ESA alone), fewer patients used IV iron along with ESA from 2006 to the second quarter of 2007. There was a notable increase in the number of patients using IV iron along with ESA as compared to ESA alone from the third quarter of 2007 to 2008. 

Of the individuals on IV iron, 85% (*n* = 5,678) received iron sucrose and the remaining 15% (*n* = 1,000) received iron dextran. Average IV iron use in CKD patients increased sharply from 2006 to 2008 (Table 6), although it still remained a small fraction of all CKD patients. 

The days of therapy (DOTs) for ESA and IV iron therapy administered to individual patients was determined. The mean duration of therapy for each drug was calculated. The aggregate of drug use for each year was expressed as DOTs per 100 patient-days (PDs).  Table 7 lists the mean DOTs/100 PDs for IV iron and ESA therapy. The mean (±SD) ESA DOTs/100 PDs was 12.36 ± 21.92 in the ESA group and 8.66 ± 20.28 in the IV iron group. *t*-test results showed a statistically significant mean difference of 3.7 [(95% CI = 3.15, 4.24), SE = 0.278, *P* < 0.001]. 

**Figure 2:**
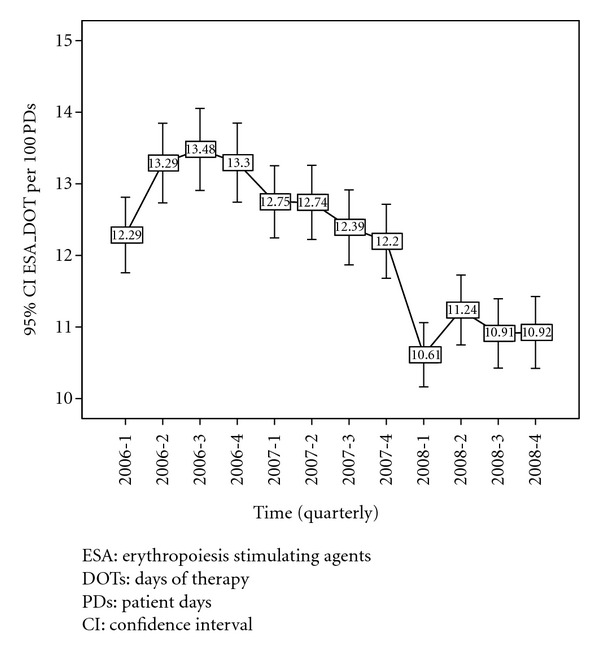
Mean ESA DOTs/100 PDs use (with 95% CI) from 2006–2008 (by each quarter).

**Figure 3:**
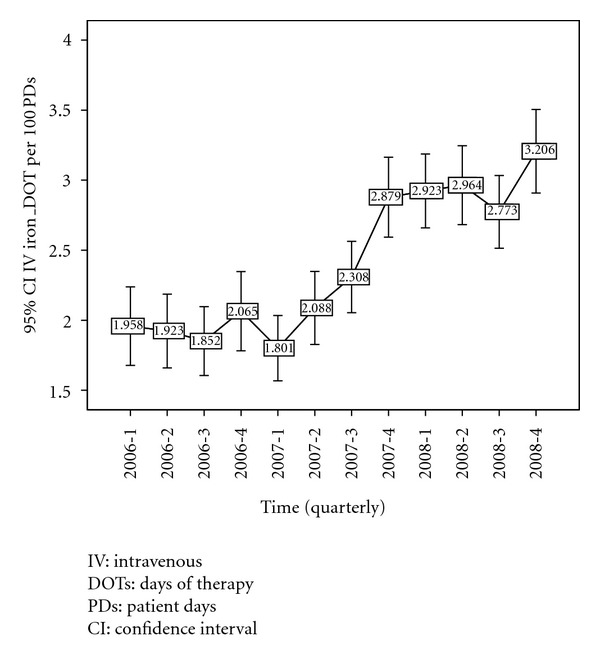
Mean IV Iron DOTs/100 PDs use (with 95% CI) from 2006–2008 (by each quarter).

Figure 2 shows change in the mean ESA DOTs/100 PDs with each quarter of drug use from 2006–2008. There is a significant increase in the mean ESA use in the first quarter of 2006 (*P* = 0.03). Notice the significant (*P* = 0.005) decrease in the use of ESA from the second quarter of 2006 to the third quarter of 2007. A substantial drop in the mean use of ESA occurred from the third quarter of 2006 to the first quarter of 2008 (*P* < 0.001).  Figure 3 shows the change in the mean IV Iron DOTs/100PDs with each quarter of drug use from 2006–2008. Note the significant increase of IV iron use from the first quarter of 2007 to the second quarter of 2008 (*P* < 0.001). 

### 3.3. Predictors of IV Iron Use

Older adults (≥65 years) were 1.246 times more likely to be prescribed IV iron for anemia of CKD compared to young adults in the age range of 18–30 years [95% CI (1.108, 1.402), *P* < 0.001]. Race was found to be a strong predictor of drug use in the anemic CKD population. African Americans and Hispanics were 0.685 [95% CI (0.647, 0.726) *P* < 0.001] times and 0.627 [95% CI (0.567, 0.695) *P* < 0.00] times as likely to receive IV iron compared to the White population on ESA therapy. Patients covered under the Medicaid, any commercial/private insurance or who paid out of pocket for insurance were 1.141 [95% CI (1.044, 1.247) *P* = 0.003], 1.265 [95% CI (1.178, 1.360) *P* < 0.001],and 1.451 [95% CI (1.171, 1.798) *P* = 0.001] times more likely to receive IV iron therapy as compared to patients covered under Medicare. 

Patient admission status, severity of illness, dialysis status, and patient length of stay were strong predictors of drug use in the anemic CKD population. Patients admitted to the hospital as emergency and elective cases were 1.34 [95%CI (1.256, 1.430) *P* < 0.001] times and 1.307 [95%CI (1.202, 1.421) *P* < 0.001] times more likely to be prescribed IV iron as compared to patients admitted to the hospital as urgent cases. “Extremely” sick CKD patients were less likely to receive IV iron as compared to “moderately” sick patients. CKD patients not on dialysis were 0.851 [95% CI (0.8, 0.907) *P* < 0.001] times as likely to receive IV iron as compared to CKD patients on dialysis. 

Nephrologists were 1.216 [95% CI (1.131, 1.308) *P* < 0.001] times more likely to prescribe IV iron to CKD patients already on ESA therapy, compared to internal medicine physicians. Transplant specialists and surgeons were 0.772 [95% CI (0.664, 0.898) *P* = 0.001] and 0.912 [95% CI (0.836, 0.995) *P* = 0.03] times as likely to prescribe IV iron to CKD patients compared to internal medicine physicians. 

## 4. Discussion

In this inquiry, we described the utilization of IV iron and ESA in anemic CKD patients. Of the 82,947 CKD patients on ESA therapy, only 8% (*n* = 6,678) were on IV iron supplementation. Of those 6678 patients on IV iron, 91% were prescribed iron sucrose, and the rest received iron dextran. Almost 30% of the CKD population was on hemodialysis (*n* = 25,322). 

Previous investigations report varying prevalences of IV iron usage. Bailie et al. found IV iron being prescribed to 20% of the patients with anemia of CKD [28]. This study included patients from four academic nephrology centers where prescribing physicians might have been more familiar with CKD treatment guidelines. Rasu et al. reported only 3% of CKD patients with anemia in outpatient settings of the US were prescribed IV iron [29]. Reasons for low prevalence of IV iron use in this investigation are unclear, particularly because the NKF-KDOQI clinical practice guidelines for anemia, which are repeatedly published since 1997, make firm recommendations for optimal use of IV iron supplementation in hemodialysis patients. 

Concerns regarding long-term safety of IV iron may have had a role in such low prevalence of use. These concerns arise from the known effect of iron as a growth factor of bacteria [30–32], its suspected inhibition of neutrophil function [33], and increased oxidative stress leading to atheromatous [34–36] change as well as anaphylactic and other adverse events associated with the use of IV iron. No large prospective clinical trials have investigated the relationship between iron dosing and infectious morbidity or mortality. Observation studies by Feldman et al. [37, 38] examined the effect of IV iron on mortality and hospitalization. One study of 32,566 hemodialysis patients during 1996 and 1997 found no significant association between cumulative iron dose and all-cause mortality. Results of this report provide cautious support for the safe use of cumulative iron doses greater than 1000 mg during 6 months, if needed to maintain target Hb levels in hemodialysis patients. However, some clinicians may still be reluctant to use iron because of lingering concerns about iron toxicity issues. 

The data indicates that ESA use is decreasing and IV iron use is increasing, although the rate of IV iron use is still quite low. Several events could explain these trends. The decrease in ESA use is likely due to safety concerns with ESA use which have resulted in a black-box warning on all ESA products instructing prescribers to use the lowest ESA dose that gradually increases hemoglobin concentrations to the lowest level sufficient to avoid the need for red blood cell transfusion [19]. Also, the FDA recently stated that all ESAs prescribed must be a part of the Risk Evaluation and Mitigation Strategies (REMS) program to ensure the safe use of these drugs [20]. 

The increase in iron use is likely due to the National Kidney Foundation-Dialysis Outcomes Quality Initiative (NKF-DOQI) of 2006 which revised the clinical practice guidelines for anemia of CKD. The NKF-KDOQI guidelines recommend that a CKD patient's hemoglobin (Hb) be checked annually regardless of the cause or state of their CKD [1]. In addition, the guidelines firmly recommend iron supplementation in order to maintain adequate iron indices that is, transferrin saturation (TSAT) and serum ferritin levels and Hb levels. 

Despite the NKF-DOQI guidelines, only 8% (*n* = 6678) of CKD patients receiving ESAs got IV iron. Of that number, most were prescribed iron sucrose. Only 9% of patients received iron dextran—most likely because of its known risk of causing anaphylactoid and other life-threatening adverse reactions. No evidence of sodium ferric gluconate use was found in this data. 

The days of therapy (DOTs) for ESA and IV iron therapy administered to individual patients was determined to quantify utilization of IV iron and ESA use, relative to the use of ESA alone. Although the defined daily dose (DDD) method is recommended by the World Health Organization to estimate drug use, important deficiencies of the defined daily dose method compared with direct measure of the DOTs have recently been reported [39–42]. Specifically, the defined daily dose method is intended to estimate the DOTs from the quantity of drug purchased by the hospital [42]. In most countries, purchase data are more readily available than measures of the DOTs. Electronic capture of pharmacy dispensing and administration data now makes it feasible to measure DOTs directly. Moreover, in the UHC data, different hospitals have different measurement of units (vial, mg, mcg, mL, units, etc.), making it difficult to quantify the dose of ESA or IV iron therapy using the DDD methodology. DDD methods will underestimate drug exposure when the administered daily dose is reduced for a patient with impaired bodily function or sudden adverse events. Also, if the administered daily dosage differs significantly from the WHO-approved DDD, then DDD methodology will not provide an accurate assessment of the number of days of therapy. Days of therapy (DOTs) are the most common alternative measure of drug consumption in hospitals. 

A clustered binomial logistic regression using generalized estimating equations (GEEs) was used to identify potential predictors of IV iron use in anemic CKD patients. Older adults (≥65 years) were significantly more likely to be prescribed IV iron for anemia of CKD compared to young adults in the age range of 18–30 years. This finding is consistent with the literature demonstrating a high prevalence of IV iron use among older adults [28]. Race was found to be a strong predictor of drug use in the anemic CKD population. Blacks and Hispanics were significantly less likely to receive IV iron compared to the White population on ESA therapy. This finding is consistent with the current literature [28, 43] and can be associated to the socioeconomic status of these races. Patients covered under the Medicaid, any commercial/private insurance or who paid out of pocket for insurance, were significantly more likely to receive IV iron therapy as compared to patients covered under Medicare. This finding is debatable considering the ambiguity in Medicare coverage decisions for ESRD and predialysis CKD patients. Dialysis patients, regardless of age, have been entitled to Medicare coverage since 1972 [44]. As a result of widespread coverage, Medicare serves as primary insurance for the majority of ESRD patients after the initiation of dialysis. Most dialysis patients below the age of 65, however, are not eligible for Medicare benefits until the fourth month after initiating dialysis. Medicare does not cover any costs of treatment during these first three months of dialysis unless the patient already has primary Medicare coverage because of age or disability. The private health plan is the only payer for the first three months of dialysis. When a patient becomes eligible for Medicare due to ESRD in the fourth month of dialysis, there is a 30-month “coordination period” when the health plan serves as the primary payer for health care services and Medicare becomes the secondary payer. At the end of this 30 month period, Medicare pays for all Medicare covered services as a primary payer, and the health plan becomes the secondary payer [44]. Only 31% of the sample CKD population (*N* = 82,947) was on dialysis, while the majority (>60%) of patients were predialysis CKD patients and in the age range of less than 65 years who may not be covered by Medicare as their primary payer. 

Patients admitted to the hospital as emergency and elective cases were significantly more likely to be prescribed IV iron as compared to patients admitted to the hospital as urgent cases. “Extremely” sick CKD patients were less likely to receive IV iron as compared to “moderately” sick patients. This finding can be explained by the concerns physicians may have regarding the use of IV iron in terminally ill patients and toxicity issues with IV iron. These concerns arise from the known effect of iron as a growth factor of bacteria [30–32], its suspected inhibition of neutrophil function [33], and increased oxidative stress leading to atheromatous [34–36] change as well as anaphylactic and other adverse events associated with the use of IV iron. CKD patients on dialysis were less likely to receive IV iron as compared to CKD patients not on dialysis. However, this finding is unusual considering that IV iron is highly recommended to CKD patients on dialysis. 

With regards to physician specialty, nephrologists were more likely to prescribe IV iron to CKD patients already on ESA therapy compared to internal medicine physicians; whereas transplant specialists and surgeons were significantly less likely to prescribe IV iron to these patients. This finding is consistent with our hypothesis. Recent emphasis has called for early referral of CKD patients to nephrologists, since this approach has been demonstrated to improve patient outcomes and result in earlier preparation for an initiation of dialysis and prescribe the appropriate therapy as needed [45]. 

Inevitably, there were limitations to this investigation. The first limitation is the difference in hospitals with respect to anemia and CKD management policies and formularies/protocols that cause variations in the utilization patterns. However, hospital level factors were used in an attempt to control for interhospital variability while assessing utilization. Also, a clustered analysis using the GEE methodology was used to account for nesting of patients within hospitals to control for erroneous inferences of associations between independent variables and drug use. In the clustered models, we put a restriction that patients within a hospital are nested and correlated when compared with patients between different hospitals. 

Adjusting for underlying severity of illness was difficult in this investigation, because there is currently no well-validated severity of illness score for CKD. Previous investigations have used a variety of techniques including the Stoke Comorbidity Grade (SCG) [46], the Khan index [47], the Davies index [48], and the Charlson comorbidity index [49]. Unlike other indices, the Davies index does not include age, because it was specifically designed to be used in conjunction with age as an independent covariate. Other comorbidity indices used in studies on ESRD patients assigned different weights to different comorbidities, such as the Khan or the Charlson index, with the weights based on the impact of comorbid diseases on survival [50]. However, the impact of comorbid diseases on survival may be different from their impact on health status and resource use [50]. This investigation used the All Patient Refined Diagnosis Related Groups (APR-DRGss) classification system to adjust for severity of underlying illness [51]. The APR-DRG system is an enhancement of the DRG structure and is a good predictor of drug utilization, hospital costs, and resource use [52, 53]. 

This investigation does not assess the appropriateness of each the patient's iron supplementation therapy. It is possible that each choice not to supplement ESA use with IV iron was rational and evidence based. However, IV iron supplementation has been linked to better patient outcomes for CKD anemia [22]. 

The patient population in this study may not represent all CKD patients in the nation. The University HealthSystem Consortium (UHC) database used for this study is derived from a group of university teaching hospitals providing tertiary care. University hospitals are different from other hospitals because they typically perform more complex procedures, have higher costs, and result in longer patient length-of-stays than community hospitals.[54] Therefore, the results of this study may not be directly extrapolated to the general hospital community of the USA 

Finally, this study faces the same limitation seen in any exploration of archived inpatient hospital data. Inaccurate coding or missing data may introduce bias in the results. Also, the database does not contain data that might be better predictors of prescribing. We were unable to assess physicians' prescribing intent or their knowledge of recommended guidelines. In addition, we could not assess pharmaceutical industry influence or laboratory values such as hemoglobin, transferrin saturation, serum ferritin, and other iron indices which may be possible predictors of IV iron use in anemic CKD patients.

## 5. Conclusion

This inquiry describes the utilization of IV iron and ESA in anemic CKD patients, with a focus on understanding predictors of drug use. Data collected from 62 teaching hospitals between 2006 and 2008 showed an increasing trend in the use of IV iron in anemic CKD patients already on ESA therapy. Use of IV iron supplementation was associated with a significant decrease in the duration of therapy of ESA. Despite this positive finding for supplementing ESA administration with IV iron, only 8% of CKD patients receiving ESAs also received IV iron. Patient demographics (age, race, primary payer), patient clinical conditions (admission status, severity of illness, dialysis status), and physician specialty were identified as predictors of IV iron use in CKD patients. 

Use rates of IV iron in this population suggest that anemia management therapies may be underutilized in CKD anemic patients in spite of emphasis on the use of IV iron in the consensus guidelines. The low treatment rate represents a gap in treatment practices and signals an opportunity for healthcare improvement. Pathologic factors, physician practice patterns, pharmaceutical industry influence affecting rates of IV iron use in anemia of CKD, along with patient-centered clinical and economic outcomes of such treatment merit further research.

## Figures and Tables

**Table 1:** Diagnoses codes (ICD-9-CM) and procedure codes used to identify Chronic Kidney Disease patients (inclusion criteria).

Diagnoses and procedure codes for inclusion criteria	
Diagnoses codes	

Description	ICD-9-CM Codes

Chronic renal failure	585.1–585.6, 585.9
Renal failure, unspecified	586
Renal sclerosis, unspecified	587
Hypertensive renal disease	403.00–403.9
Hypertensive heart and renal disease	404.00–404.9
Nephrotic syndrome	581.0–581.9
Chronic glomerulonephritis	582.0–582.9
Nephritis (NOS as acute or chronic)	583.0–583.9
Chronic pyelonephritis (without lesion of renal medullary necrosis)	590.00
Chronic pyelonephritis (with lesion of renal medullary necrosis)	590.01
Renal dialysis status	V45.1
Fitting or adjustment to dialysis catheter	V56.1-V56.2
Adequacy testing for hemodialysis or peritoneal dialysis	V56.3, V56.31, V56.32
Encounter for dialysis and dialysis catheter care	V56.0, V56.8
Anemia of Chronic kidney disease	285.21

Procedure codes	

Hemodialysis	39.95
Peritoneal dialysis	54.98

**Table 2:** Diagnoses codes (ICD-9-CM) and procedure codes used to identify Chronic Kidney Disease patients (exclusion criteria).

Diagnoses and procedure codes for exclusion criteria	
Diagnoses codes	

Description	ICD-9-CM Codes

Neoplasms	140.00–239.00
Blood transfusion	V58.2
Kidney/other organ transplant	996.8, E878.0, V42
Gastrointestinal bleeding	569.3, 578.9, 626, 627
HIV/AIDS	042, V08, 795.71

Procedure codes	

Chemotherapy	00.10, 99.85, 99.25, 92.28, 99.28
Radiation therapy	14.26, 92.41, 92.25, 92.21, 92.22, 0.18, 14.27, 92.26
Blood transfusion	99.03, 38.92, 38.94, 99.02
Kidney/organ transplant	00.91–00.93
Gastrointestinal bleeding	44.43, 44.44

**Table 3:** Demographic characteristics of the study sample.

Variable	ESA + IV Iron	ESA alone	Total
	*n* = 6678	*n* = 76269	*N* = 82947
Demographic characteristics			

	*n* (%)	*n* (%)	*N* (%)

Age group (years)*			
18–30	369 (5.53)	4728 (6.20)	5097 (6.14)
31–50	1476 (22.10)	19349 (25.37)	20825 (25.11)
51–64	2074 (31.06)	24546 (32.18)	26620 (32.09)
≥65	2759 (41.31)	27647 (36.25)	30406 (36.66)

Race*			
White	3348 (50.13)	30504 (40)	33852 (40.81)
Black	2299 (34.43)	32010 (41.97)	34310 (41.36)
Hispanic	514 (7.70)	7742 (10.15)	8256 (9.95)
Other	517 (7.74)	6013 (7.88)	6530 (7.87)

Gender			
Male	3615 (54.13)	39940 (52.37)	43555 (52.51)
Female	3063 (45.87)	36330 (47.63)	39393 (47.49)

Primary payer*			
Commercial/Private payer	1157 (17.33)	11092 (14.54)	12249 (14.77)
Medicare	4588 (68.70)	53834 (70.58)	58422 (70.43)
Medicaid	710 (10.63)	9026 (11.83)	9736 (11.74)
Self-pay	97 (1.45)	924 (1.21)	1021 (1.23)
Other	126 (1.89)	1394 (1.83)	1520 (1.83)

*Differences between the study groups were statistically significance at *P* < 0.05.

**Table 4:** Clinical characteristics of the study sample.

Variable	ESA + IV Iron	ESA alone	Total
	*n* = 6678	*n* = 76269	*N* = 82947
Clinical characteristics			

Admission status*			
Emergency	4130 (61.84)	44360 (58.16)	48490 (58.46)
Urgent	1436 (21.50)	20015 (26.24)	21451 (25.86)
Elective	1017 (15.23)	10636 (13.95)	11653 (14.05)
Other	95 (1.42)	1259 (1.65)	1354 (1.63)

Severity of illness*			
Moderate	817 (12.32)	10205 (13.38)	11022 (13.29)
Major	3859 (57.79)	41673 (54.64)	45532 (54.89)
Extreme	2002 (29.98)	24392 (31.98)	26394 (31.82)

Discharge status*			
Discharged/Transferred alive	6440 (96.44)	72782 (95.43)	79222 (95.51)
Expired	236 (3.53)	3461 (4.54)	3697 (4.46)
Other	2 (0.03)	26 (0.03)	28 (0.03)

Dialysis status*			
On dialysis	1776 (26.59)	23546 (30.87)	25322 (30.53)
Not on dialysis	4902 (73.41)	52724 (69.13)	57626 (69.47)

Physician specialty*			
Internal medicine	2182 (32.67)	25652 (33.63)	27834 (33.56)
Nephrology	1319 (19.75)	12793 (16.77)	14112 (17.01)
Cardiology	750 (11.23)	8141 (10.67)	8891 (10.72)
Transplant	273 (4.09)	3696 (4.85)	3969 (4.78)
Pulmonary/Critical care	196 (2.94)	2635 (3.45)	2831 (3.41)
Hospitalist	320 (4.79)	3391 (4.45)	3711 (4.47)
Surgery	823 (12.32)	9260 (12.14)	10083 (12.16)

Bed size*			
1–399	1728 (25.88)	12309 (16.14)	14037 (16.92)
400–599	1281 (19.18)	22099 (28.98)	23380 (28.19)
600–799	2005 (30.02)	25584 (33.54)	27589 (33.26)
800 or more	1664 (24.92)	16278 (21.34)	17942 (21.63)

Geographical region*			
Midwest	2416 (36.18)	17498 (22.94)	19914 (24.01)
Northeast	1826 (27.34)	17045 (22.35)	18871 (22.75)
Southeast	848 (12.70)	17264 (22.64)	18112 (21.84)
Southwest	456 (6.83)	10879 (14.26)	11335 (13.67)
West	1132 (16.95)	13583 (17.81)	14715 (17.74)

*Differences between the study groups were statistically significance at *P* < 0.05.

**Table 5:** Treatment characteristics of the study sample.

Variable	ESA + IV Iron	ESA alone	Total
	*n* = 6678	*n* = 76269	*N* = 82947
Treatment characteristics			

ESA type*			
Epoetin	3428 (51.3)	44106 (57.8)	47534 (57.3)
Darbepoetin	2082 (31.2)	32163 (42.2)	34245 (41.3)
Unknown	1168 (17.5)	0 (0)	1168 (1.4)

IV iron type			
Iron sucrose	5678 (85)	NA	NA
Iron dextran	1000 (15)		

*Differences between the study groups were statistically significance at *P* < 0.05.

**Table 6:** Annual percentages of chronic kidney disease patients treated with IV iron.

	2006	2007	2008
*Hemodialysis *			
Total number of patients	395	544	779
Iron sucrose (%)	359 (91)	514 (94)	718 (92)
Iron dextran (%)	36 (9)	30 (6)	61 (8)

*Peritoneal dialysis*			
Total number of patients	17	18	23
Iron sucrose (%)	10 (41)	16 (89)	19 (83)
Iron dextran (%)	7 (59)	2 (11)	4 (17)

*Not on dialysis*			
Total number of patients	1197	1516	2189
Iron sucrose (%)	923 (77)	1263 (83)	1856 (85)
Iron dextran (%)	274 (23)	253 (17)	333 (15)

**Table 7:** Mean days of therapy/100 patient days by drug group.

Variable	IV Iron +ESA therapy		ESA therapy alone		
	Mean	SD	Mean	SD	*P*-value
ESA DOTs/100 PDs	8.66	20.28	12.36	21.92	<0.001
IV Iron DOTs/100 PDs	30.41	28.20	NA	NA	NA

IV: Intravenous.

ESA: Erythropoiesis stimulating agents.

DOTs: Days of therapy.

PDs: Patient days.
